# Membrane Oxygenation Improves Functional Myocardial Preservation and Enables Colloid-Enriched Perfusion in the Langendorff Isolated Heart Model

**DOI:** 10.3390/medsci14030361

**Published:** 2026-06-30

**Authors:** Vasileios Leivaditis, Francesk Mulita, Athanasios Papatriantafyllou, Elias Liolis, Ioannis Panagiotopoulos, Manfred Dahm, Dimitrios Dougenis, Efstratios Koletsis

**Affiliations:** 1 Department of Cardiothoracic and Vascular Surgery, Westpfalz Klinikum, 67655 Kaiserslautern, Germany; apapatriantafy@westpfalz-klinikum.de (A.P.); mdahm@westpfalz-klinikum.de (M.D.); 2 Department of General Surgery, General University Hospital of Patras, 26504 Patras, Greece; 3 Department of Oncology, General University Hospital of Patras, 26504 Patras, Greece; lioliselias@yahoo.gr; 4 Department of Cardiac Surgery, Ippokrateio General Hospital of Athens, 11527 Athens, Greece; mdgiapan@yahoo.gr; 5 Department of Cardiac Surgery, Attikon University Hospital of Athens, 12461 Athens, Greece; ddougen@gmail.com; 6 Department of Cardiothoracic Surgery, General University Hospital of Patras, 26504 Patras, Greece; ekoletsis@hotmail.com

**Keywords:** Langendorff model, isolated heart perfusion, membrane oxygenator, extracorporeal membrane oxygenation, bovine serum albumin, myocardial preservation, ischemia–reperfusion injury, coronary flow

## Abstract

**Background**: The Langendorff isolated heart model remains one of the most widely used experimental platforms for cardiovascular research. However, conventional bubble oxygenation is associated with several limitations, including inefficient gas utilization and incompatibility with protein-containing perfusates due to excessive foam formation. The present study evaluated whether membrane oxygenation could improve myocardial preservation and facilitate the use of a protein-enriched perfusion solution in a constant-pressure Langendorff system. **Methods**: A total of 48 male Wistar rats were allocated to six experimental groups (*n* = 8 per group). In the first experimental series, myocardial performance was compared between a conventional bubble oxygenator, a Terumo CAPIOX^®^ FX05 membrane oxygenator, and a Novalung iLA membrane oxygenator. In the second series, standard Krebs–Henseleit buffer was compared with a bovine serum albumin-enriched perfusate under membrane oxygenation. Hemodynamic parameters, coronary flow, and perfusate pH were assessed throughout a 180 min ischemia–reperfusion protocol. **Results**: Both membrane oxygenators demonstrated significantly improved myocardial preservation compared with the conventional bubble oxygenator, as evidenced by superior systolic and diastolic function, enhanced coronary flow, and improved overall cardiac performance. No significant differences were observed between the two membrane oxygenators. Membrane oxygenation additionally enabled stable supplementation of the perfusate with bovine serum albumin, which resulted in further improvements in ventricular function and coronary perfusion. Perfusate pH remained comparable among groups. Furthermore, membrane oxygenation reduced Carbozen consumption by approximately 33%, increasing the number of experiments that could be performed using a standard gas cylinder. **Conclusions**: The present findings suggest that membrane oxygenation may represent a simple and effective refinement of the Langendorff isolated heart model. Beyond improving myocardial preservation, it enables the use of protein-enriched perfusates and substantially reduces gas consumption. These findings support the incorporation of membrane oxygenation into modern Langendorff systems and provide a foundation for the development of more physiologically relevant isolated organ perfusion models.

## 1. Introduction

The isolated perfused heart remains one of the most important experimental models in cardiovascular research. Since the original description of retrograde coronary perfusion by Langendorff in 1895, the technique has undergone numerous technical refinements while preserving its fundamental principle of maintaining cardiac viability ex vivo through aortic perfusion of the coronary circulation [1,2,3,4]. More than a century after its introduction, the Langendorff model continues to represent a cornerstone of experimental cardiology and is extensively used for the investigation of myocardial physiology, ischemia–reperfusion injury, coronary vascular function, electrophysiology, pharmacological interventions, and cardioprotective strategies [2,3,4,5,6].

A major advantage of the Langendorff preparation is the ability to study the heart under highly controlled conditions while eliminating systemic neurohumoral, endocrine, and metabolic influences. Parameters such as perfusion pressure, coronary flow, temperature, oxygenation, substrate composition, and pharmacological treatment can be precisely manipulated, allowing detailed evaluation of myocardial function and direct assessment of therapeutic interventions [2,4,5]. For this reason, the isolated heart model remains widely utilized in contemporary cardiovascular research despite the development of increasingly sophisticated in vivo and ex vivo experimental platforms [5,6].

Nevertheless, important limitations of conventional Langendorff systems persist. Most isolated heart preparations continue to employ crystalloid Krebs–Henseleit-based perfusion solutions oxygenated through direct gas bubbling within the perfusate reservoir. Although this approach is technically simple and inexpensive, it may provide non-uniform gas exchange and introduce gas directly into the perfusion circuit. Furthermore, bubble oxygenation significantly restricts modification of the perfusate composition because the addition of proteins or other colloidal substances results in excessive foam formation, instability of the perfusion system, and a potential risk of air embolization.

Another important limitation of conventional crystalloid perfusion is the absence of physiological colloid osmotic pressure. Standard Krebs–Henseleit solutions are protein-free and therefore lack the oncotic properties of blood. As a consequence, prolonged isolated heart perfusion promotes transcapillary fluid extravasation and progressive accumulation of interstitial myocardial edema. Myocardial edema has been associated with impaired ventricular compliance, increased diastolic stiffness, deterioration of coronary microcirculatory function, and progressive reduction in myocardial performance during prolonged perfusion experiments [7,8,9,10]. This phenomenon may limit both the duration of experiments and the physiological relevance of the obtained results.

An additional consideration is that myocardial performance in crystalloid-perfused isolated heart preparations may be fundamentally limited by oxygen transport capacity. Although Krebs–Henseleit buffer provides adequate substrate delivery and allows stable short-term perfusion, several studies have suggested that oxygen delivery to the myocardium may become suboptimal during prolonged ex vivo perfusion, particularly under conditions of increased metabolic demand. Unlike blood, crystalloid perfusates lack hemoglobin and therefore rely exclusively on dissolved oxygen for tissue oxygenation. As a result, oxygen transport capacity is substantially lower than under physiological conditions, potentially limiting mitochondrial oxygen availability despite apparently adequate perfusate oxygenation. Kuzmiak-Glancy et al. demonstrated that cardiac performance in crystalloid-perfused hearts is constrained by oxygen delivery to mitochondria and that myocardial energetics remain closely linked to oxygen transport limitations even under standard Langendorff conditions [11]. Similar observations have been reported by previous investigators, who identified oxygen diffusion and delivery as important determinants of myocardial function and metabolic stability in isolated heart preparations [12,13,14,15]. These findings suggest that optimization of oxygen transfer within the perfusion circuit may represent an important, yet often underappreciated, determinant of myocardial preservation and experimental reproducibility.

In contrast to conventional bubble oxygenators, membrane oxygenators have become the standard method of extracorporeal gas exchange in modern cardiopulmonary bypass and extracorporeal life support systems because of their superior gas transfer efficiency, reduced gaseous microembolization, and improved biocompatibility [16,17,18]. Despite their widespread clinical application, membrane oxygenators have received relatively limited attention in isolated heart perfusion models. The incorporation of membrane oxygenation into a Langendorff system may offer several theoretical advantages, including more stable oxygen delivery, elimination of direct gas bubbling, improved control of perfusate composition, and the possibility of introducing protein-containing perfusion solutions without foam formation.

The ability to supplement isolated heart perfusates with proteins may be particularly important. Restoration of colloid osmotic pressure may reduce interstitial fluid accumulation and thereby contribute to improved myocardial preservation during prolonged perfusion, although this mechanism was not directly evaluated in the present study. Such an approach may provide a more physiological ex vivo environment and enhance the functional stability of isolated hearts subjected to ischemia–reperfusion injury.

Our group has previously utilized the constant-pressure Langendorff model extensively for the investigation of myocardial ischemia–reperfusion injury and cardioprotective pharmacological interventions [19]. Building upon this experience, we sought to further optimize the technical characteristics of the isolated heart perfusion system itself.

Therefore, the aim of the present study was first to compare a conventional bubble oxygenator with two membrane oxygenator systems—a pediatric cardiopulmonary bypass membrane oxygenator and an extracorporeal lung assist (ECLA) membrane oxygenator—in a constant-pressure isolated rat heart ischemia–reperfusion model. Following establishment of the superiority of membrane oxygenation, a second series of experiments was performed to investigate whether supplementation of the perfusate with protein could further improve myocardial functional preservation. We hypothesized that membrane oxygenation would enhance myocardial performance and enable the use of colloid-enriched perfusates, potentially providing a more physiologically relevant and functionally stable Langendorff perfusion model.

## 2. Materials and Methods

### 2.1. Animal Cohort

The study was approved by the Institutional Committee for Animal Care and Use of the University of Patras and the Prefecture of Western Greece (Protocol Nr. 261936/1076; date of approval 28 September 2017).

A total of 48 male Wistar rats (20–24 weeks old, body weight 350–400 g) were included in the study. All animals received humane care in accordance with European legislation governing the use of animals in scientific research (Directive 2010/63/EU) and the Consensus Author Guidelines on Animal Ethics and Welfare for Veterinary Journals. Animals were housed one to two per cage under standardized laboratory conditions, including controlled temperature and humidity and a 12 h light/12 h dark cycle, with unrestricted access to standard rodent chow and water.

Sample size estimation was performed using dedicated power analysis software (version 3, Power and Sample Size Calculation, powerandsamplesize.com). Based on a statistical power of 80%, a significance level (α) of 0.05, and the expected differences in hemodynamic parameters between the experimental and control groups, the required sample size was calculated to be eight animals per group. The animals were randomly assigned to six experimental groups (*n* = 8 per group).

### 2.2. Isolated Heart Preparation

The Langendorff isolated heart preparation is a well-established and highly reproducible experimental model for the assessment of myocardial contractile function and the investigation of responses to ischemia–reperfusion injury [20,21,22,23]. The isolated heart experiments were performed using a constant-pressure Langendorff perfusion system.

Animals were anesthetized with pentobarbital sodium (50 mg/kg, intraperitoneally), ketamine (75 mg/kg, intraperitoneally) and xylazine (10 mg/kg, intraperitoneally). Following median laparotomy, the inferior vena cava was exposed and systemic anticoagulation was achieved by administration of heparin sodium (1000 IU/kg) to prevent intravascular clot formation.

Following median sternotomy, the hearts were rapidly excised and immersed in ice-cold Krebs–Henseleit buffer (KHB) to arrest mechanical activity and minimize ischemic injury. The ascending aorta was cannulated and connected to the perfusion apparatus for retrograde aortic perfusion.

Perfusion was performed at a constant pressure of 75 mmHg using KHB consisting of 120 mM NaCl, 25 mM NaHCO_3_, 5.9 mM KCl, 1.2 mM MgSO_4_, 2.25 mM CaCl_2_, 1.2 mM KH_2_PO_4_ and 11 mM glucose [21,22,23,24].

The perfusate temperature was maintained at 38 °C using a thermostatically controlled water-circulating system. Throughout the experiments, the pH of the perfusate was maintained at approximately 7.4.

**Figure 1:**
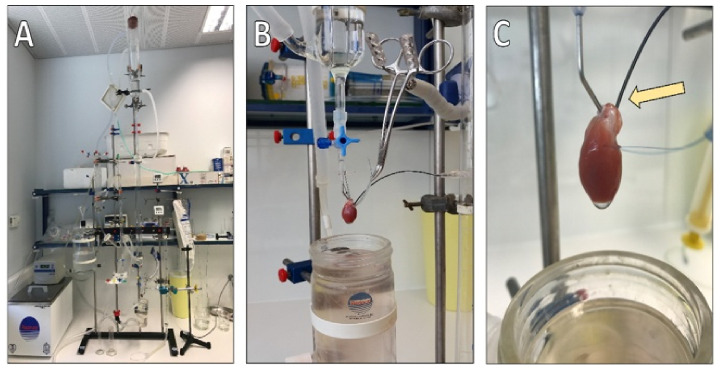
Constant-pressure Langendorff isolated heart perfusion system. (**A**) Overview of the experimental Langendorff apparatus used for retrograde aortic perfusion of isolated rat hearts. (**B**) Isolated rat heart mounted on the perfusion system following aortic cannulation. (**C**) Close-up view of the intraventricular latex balloon inserted through the left atrium (arrow) into the left ventricle for continuous assessment of left ventricular pressure and derivation of hemodynamic parameters including LVSP, LVEDP, LVDP, and ±dP/dt.

A thin latex balloon connected to a pressure transducer was introduced into the left ventricle through the left atrium and mitral valve. The balloon volume was adjusted to achieve an initial left ventricular end-diastolic pressure (LVEDP) of approximately 5 mmHg before the beginning of the experimental protocol. A representative overview of the constant-pressure Langendorff apparatus, the isolated heart preparation, and the intraventricular balloon positioning used for hemodynamic measurements is presented in Figure 1.

### 2.3. Oxygenation Systems

Three different oxygenation systems were evaluated.

- Conventional Bubble Oxygenator: In the conventional setup, the perfusate was oxygenated using a standard bubble oxygenator (Radnoti LLC, Monrovia, CA, USA). A Carbozen gas mixture consisting of 95% O_2_ and 5% CO_2_ was continuously introduced into the oxygenator, allowing direct gas–liquid contact and oxygenation of the KHB solution.
- Terumo CAPIOX^®^ FX05 Membrane Oxygenator: In the second configuration, a pediatric hollow-fiber membrane oxygenator (CAPIOX^®^ FX05, Terumo Cardiovascular Systems, Tokyo, Japan) was incorporated into the perfusion circuit. Gas exchange occurred through the membrane fibers without direct contact between the gas phase and the perfusate.
- Novalung iLA^®^ Membrane Oxygenator: In the third configuration, an extracorporeal lung assist membrane oxygenator (iLA^®^ Membrane Ventilator, Novalung GmbH, Heilbronn, Germany) was integrated into the circuit using the same gas mixture and operating principles.

**Figure 2:**
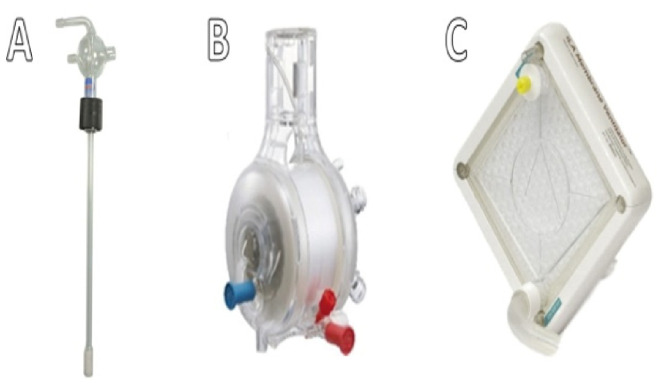
Comparison of the oxygenation systems used in the experimental Langendorff model. (**A**) Conventional bubble oxygenator (Radnoti LLC, Monrovia, CA, USA), in which oxygenation is achieved through direct gas–liquid contact. (**B**) Pediatric membrane oxygenator CAPIOX^®^ FX05 (Terumo Cardiovascular Systems Corporation, Tokyo, Japan). (**C**) Extracorporeal lung assist membrane oxygenator iLA^®^ Membrane Ventilator (Novalung GmbH, Heilbronn, Germany). The membrane oxygenators were incorporated into the perfusion circuit to provide indirect gas exchange via semipermeable hollow fibers, thereby eliminating direct bubbling of the perfusate and permitting the use of protein-enriched perfusion solutions.

For both membrane oxygenators, oxygenation was achieved using Carbozen (95% O_2_ and 5% CO_2_). A representative overview of the oxygenation systems used in the present study, including the conventional bubble oxygenator and the two membrane oxygenators, is presented in Figure 2.

Before initiation of the experimental series, preliminary system standardization experiments were performed to determine the gas flow rates required to achieve adequate oxygenation of the perfusate for each oxygenation system. During this setup phase, perfusate pO_2_ and oxygen saturation were intermittently assessed using blood gas analysis. These measurements were used to confirm adequate oxygenation and to identify the minimum gas flow required for stable perfusion conditions. However, these assessments were performed as part of system optimization and were not prospectively collected for all experimental groups or individual experiments. Consequently, these data were not included in the formal statistical analysis of the study.

### 2.4. Experimental Groups

The first experimental series was designed to compare the different oxygenation systems.

- Group 1 (Conventional Oxygenator): Hearts subjected to ischemia–reperfusion injury and perfused using the conventional bubble oxygenator.
- Group 2 (CAPIOX FX05): Hearts subjected to ischemia–reperfusion injury and perfused using the Terumo membrane oxygenator.
- Group 3 (Novalung iLA): Hearts subjected to ischemia–reperfusion injury and perfused using the Novalung membrane oxygenator.
- Group 4 (Sham): Hearts continuously perfused without induction of ischemia.

Following completion of the oxygenator comparison experiments, a second experimental series was performed using membrane oxygenation.

- Group 5 (KHB): Hearts perfused with standard Krebs–Henseleit buffer using membrane oxygenation.
- Group 6 (KHB + BSA): Hearts perfused with bovine serum albumin (BSA)-enriched Krebs–Henseleit buffer using membrane oxygenation.

The two experimental series were conducted using separate animal cohorts. Groups 5 and 6 were therefore completely independent from Groups 1–4, and no isolated heart preparation was included in more than one experimental group. This design was chosen to ensure methodological consistency and to avoid potential carryover effects between experimental interventions. For the second experimental series, the CAPIOX^®^ FX05 membrane oxygenator was used in both Groups 5 and 6. The selection of this oxygenator was not based on evidence of superiority over the Novalung iLA^®^ membrane oxygenator, as no significant differences were observed between the two membrane oxygenation systems in the first experimental series. Rather, a single membrane oxygenator was used for both groups to ensure consistency when evaluating the effects of perfusate composition.

The rationale for the second experimental series was based on the observation that direct gas bubbling caused extensive foam formation in protein-containing perfusates, making the use of colloid-enriched solutions impractical with conventional oxygenators. Membrane oxygenation completely eliminated this problem and allowed stable perfusion with protein-containing solutions.

The concentration of bovine serum albumin was selected based on previous experience with isolated organ perfusion systems and on its widespread use in experimental perfusion protocols. A concentration of 1% (10 g/L) provides a substantial increase in colloid osmotic pressure compared with standard crystalloid Krebs–Henseleit buffer while maintaining low perfusate viscosity and preserving stable perfusion conditions.

A concentration of 1% bovine serum albumin was selected as a proof-of-concept intervention to evaluate whether partial restoration of colloid osmotic pressure could improve myocardial preservation during membrane-oxygenated perfusion. The objective was not to determine the optimal albumin concentration but rather to assess the feasibility and physiological effects of protein supplementation within the modified Langendorff system. Plasma albumin concentrations are approximately 35–50 g/L (3.5–5%), but reproducing full plasma oncotic pressure was beyond the scope of the study. We selected a moderate concentration that could increase colloid osmotic pressure while maintaining a simple and reproducible crystalloid perfusate. Furthermore, this concentration has been employed in several ex vivo organ perfusion studies and represents a practical compromise between physiological relevance and technical feasibility [25,26,27,28].

The aim of the present study was not to determine the optimal albumin concentration but rather to investigate whether the incorporation of a physiologically relevant protein component into the perfusate could provide functional benefits when membrane oxygenation was used.

### 2.5. Experimental Protocol

After mounting on the Langendorff apparatus, all hearts underwent a stabilization period of 30 min. Regional myocardial ischemia was then induced by ligation of the left anterior descending coronary artery (LAD). A 6-0 polypropylene suture was passed beneath the LAD close to its origin and threaded through a small plastic tourniquet. Tightening of the tourniquet produced coronary occlusion, whereas release of the ligature initiated reperfusion.

The experimental protocol consisted of:

- 30 min stabilization
- 30 min regional ischemia
- 120 min reperfusion

**Figure 3:**
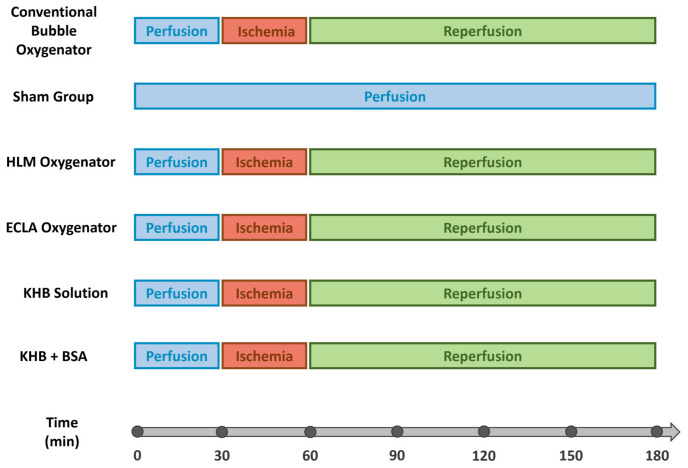
Schematic representation of the experimental protocol. All isolated hearts underwent a 30 min stabilization period followed by 30 min of regional myocardial ischemia induced by left anterior descending coronary artery (LAD) occlusion and 120 min of reperfusion. Hemodynamic parameters and coronary flow were assessed throughout the experimental period. Sham hearts were continuously perfused and were not subjected to ischemia.

The Sham group underwent continuous perfusion without LAD occlusion. A schematic overview of the experimental protocol is presented in Figure 3.

### 2.6. Hemodynamic Measurements

Left ventricular pressure was continuously recorded throughout the experiment.

The following hemodynamic parameters were calculated:

- Left ventricular systolic pressure (LVSP)
- Left ventricular end-diastolic pressure (LVEDP)
- Left ventricular developed pressure (LVDP = LVSP − LVEDP)
- Heart rate (HR)
- Rate pressure product (RPP = HR × LVSP)
- Maximum positive first derivative of pressure (+dP/dt)
- Maximum negative first derivative of pressure (−dP/dt)

Measurements were recorded at baseline, during ischemia and at predefined intervals throughout reperfusion. LVDP was calculated as the difference between LVSP and LVEDP, while RPP was calculated as the product of heart rate and LVSP. Left ventricular pressure measurements were obtained using a custom-made intraventricular balloon connected to a disposable pressure transducer and pressure amplifier (NL108A, Digitimer Ltd., Hertfordshire, UK). The amplified signal was digitized using a LabJack U3HV data acquisition system (LabJack Corporation, Lakewood, CO, USA) at a sampling rate of 500 Hz.

Before each experiment, the pressure measurement system was calibrated using a water column over a pressure range of 10–50 cmH_2_O. Following calibration, the balloon was inserted into the left ventricle through the left atrium and connected to the recording system. Pressure signals were continuously acquired until completion of the experimental protocol.

Data processing and analysis were performed using MATLAB software (Version 26.1, The MathWorks Inc., Natick, MA, USA). Raw pressure recordings were processed to derive the corresponding hemodynamic parameters. Measurements were analyzed at 12 predefined time points throughout the experiment (15, 30, 45, 60, 75, 90, 105, 120, 135, 150, 165, and 180 min).

### 2.7. Coronary Flow

Coronary flow was assessed by collecting the coronary effluent emerging from the venous drainage of the isolated heart preparation. Effluent was collected for 2 min at 12 predefined time points during the experiment (15, 30, 45, 60, 75, 90, 105, 120, 135, 150, 165, and 180 min). The volume of collected perfusate was measured, and coronary flow was calculated and expressed as milliliters per minute (mL/min).

### 2.8. pH Measurements

Samples of the perfusate were collected at predefined time points and analyzed using a blood gas analyzer. These measurements were used to evaluate the stability and efficiency of oxygenation achieved by the different oxygenator systems.

### 2.9. Statistical Analysis

Continuous variables are presented as mean ± standard deviation (SD). Longitudinal mixed-effects regression models were used to assess the effects of time and experimental group on the measured parameters. Pairwise comparisons between groups were performed using Bonferroni-adjusted post hoc testing. Statistical significance was defined as a two-sided *p*-value below 0.05. Because the pairwise comparisons and the longitudinal mixed-effects regression analyses address different statistical questions, *p*-values may differ between the corresponding tables. Pairwise comparisons evaluate adjusted differences between groups after correction for multiple testing, whereas mixed-effects models assess the overall effect of group assignment across all repeated measurements while accounting for within-subject temporal variation. All statistical analyses were performed using STATA version 14 (StataCorp LLC, College Station, TX, USA).

## 3. Results

All isolated hearts successfully completed the experimental protocol and were included in the final analysis. The effects of the different oxygenation strategies and perfusate compositions on myocardial performance were evaluated using longitudinal mixed-effects regression models and pairwise group comparisons.

Compared with the conventional bubble oxygenator, membrane oxygenation was associated with significantly improved preservation of myocardial function across multiple hemodynamic parameters and coronary flow. No significant differences were observed between the two membrane oxygenator systems. Following these findings, a second experimental series was performed to evaluate the effects of bovine serum albumin supplementation during membrane oxygenation. Compared with standard Krebs–Henseleit buffer, albumin-enriched perfusion was associated with further improvements in several measures of cardiac performance and coronary flow.

The results of pairwise comparisons between groups are summarized in Table 1. Longitudinal mixed-effects regression analyses comparing the conventional bubble oxygenator with the membrane oxygenator groups are presented in Table 2, direct comparisons between the two membrane oxygenators in Table 3, and the effects of protein supplementation in Table 4. The regression coefficients and *p*-values presented in Table 2, Table 3 and Table 4 are derived from longitudinal mixed-effects models and are therefore not directly comparable to the Bonferroni-adjusted pairwise comparisons presented in Table 1.

Table 2 presents the longitudinal mixed-effects regression analysis comparing the conventional bubble oxygenator with the two membrane oxygenator systems and the sham group.

Table 3 summarizes the direct comparison between the CAPIOX^®^ FX05 membrane oxygenator and the Novalung iLA^®^ membrane oxygenator.

Table 4 presents the longitudinal analysis evaluating the effects of protein supplementation of the perfusate during membrane oxygenation.

Taken together, these findings indicate that membrane oxygenation is associated with substantially improved functional preservation in the Langendorff model and enables the successful use of protein-enriched perfusates.

### 3.1. Conventional Bubble Oxygenator vs. Membrane Oxygenators

#### 3.1.1. Left Ventricular Systolic Pressure (LVSP)

Left ventricular systolic pressure (LVSP) is a key indicator of myocardial contractile performance and reflects the ability of the left ventricle to generate pressure during systole. Higher LVSP values are generally associated with improved preservation of myocardial function following ischemia–reperfusion injury [2,3,29].

The longitudinal analysis demonstrated significantly higher LVSP values in hearts perfused using membrane oxygenation compared with the conventional bubble oxygenator. Both the CAPIOX FX05 membrane oxygenator group (MD = 13.78 mmHg, *p* < 0.001) and the ECLA Oxygenator group (MD = 13.75 mmHg, *p* < 0.001) exhibited significantly higher LVSP throughout the experimental period than the Conventional Oxygenator group. In contrast, no statistically significant difference was observed between the two membrane oxygenator systems (MD = 0.03 mmHg, *p* > 0.999).

**Figure 4:**
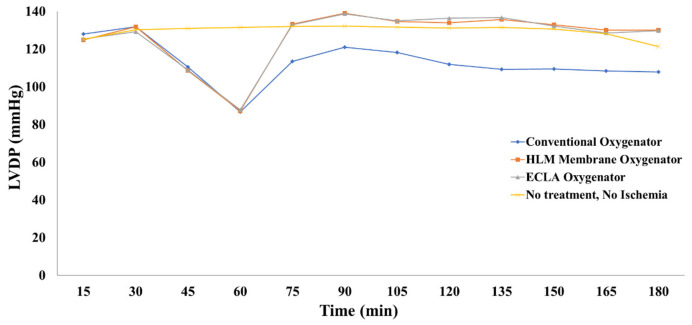
Changes in LVSP across groups over time.

The temporal evolution of LVSP in the experimental groups is illustrated in Figure 4. Hearts perfused with membrane oxygenators demonstrated superior preservation of systolic function throughout reperfusion compared with hearts perfused using the conventional bubble oxygenator.

#### 3.1.2. Left Ventricular End-Diastolic Pressure (LVEDP)

Left ventricular end-diastolic pressure (LVEDP) is an important indicator of diastolic ventricular function and myocardial compliance. Following ischemia–reperfusion injury, elevated LVEDP is generally associated with impaired ventricular relaxation, increased myocardial stiffness, and poorer preservation of cardiac function. Consequently, lower LVEDP values reflect improved diastolic performance and better overall myocardial recovery [3,29].

The longitudinal analysis demonstrated significantly lower LVEDP values in hearts perfused using membrane oxygenation compared with the conventional bubble oxygenator. Both the CAPIOX FX05 membrane oxygenator group (MD = −5.27 mmHg, *p* < 0.001) and the ECLA Oxygenator group (MD = −5.02 mmHg, *p* < 0.001) exhibited significantly lower LVEDP throughout the experimental period than the Conventional Oxygenator group. No statistically significant difference was observed between the two membrane oxygenator systems (MD = −0.26 mmHg, *p* > 0.999).

**Figure 5:**
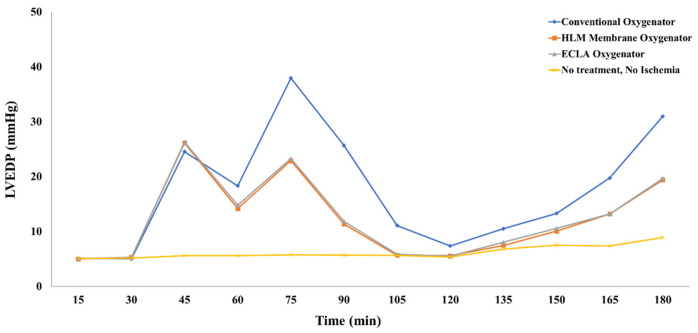
Changes in LVEDP across groups over time.

The temporal evolution of LVEDP in the experimental groups is presented in Figure 5. Hearts perfused using membrane oxygenators demonstrated improved preservation of diastolic function during reperfusion, as evidenced by the significantly lower LVEDP values compared with the conventional bubble oxygenator group.

#### 3.1.3. Left Ventricular Developed Pressure (LVDP)

Left ventricular developed pressure (LVDP) represents the difference between left ventricular systolic pressure and left ventricular end-diastolic pressure and is widely regarded as one of the most comprehensive indicators of overall myocardial performance in isolated heart experiments. Higher LVDP values reflect superior preservation of both systolic contractility and diastolic function following ischemia–reperfusion injury [3,22].

The longitudinal analysis demonstrated significantly higher LVDP values in hearts perfused using membrane oxygenation compared with the conventional bubble oxygenator. The CAPIOX FX05 membrane oxygenator group (MD = 19.05 mmHg, *p* < 0.001) and the ECLA Oxygenator group (MD = 18.77 mmHg, *p* < 0.001) both exhibited significantly greater LVDP throughout the experimental period than the Conventional Oxygenator group. No statistically significant difference was observed between the two membrane oxygenator systems (MD = 0.28 mmHg, *p* > 0.999).

**Figure 6:**
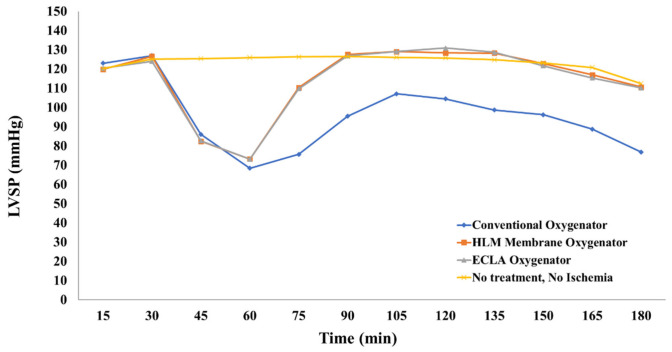
Changes in LVDP across groups over time.

The temporal changes in LVDP are presented in Figure 6. Hearts perfused with membrane oxygenators demonstrated markedly improved preservation of ventricular function during reperfusion compared with hearts perfused using the conventional bubble oxygenator. The comparable performance of the HLM and ECLA membrane oxygenators suggests that the observed functional benefit may be related to the use of membrane oxygenation itself rather than to characteristics of a specific device.

#### 3.1.4. Heart Rate (HR)

Heart rate (HR) reflects the intrinsic chronotropic activity of the isolated heart and provides an indirect measure of myocardial viability following ischemia–reperfusion injury. Preservation of heart rate during reperfusion is generally indicative of improved recovery of cardiac electrical activity and overall functional integrity [3,6,30].

The longitudinal analysis demonstrated significantly higher heart rates in hearts perfused using membrane oxygenation compared with the conventional bubble oxygenator. Both the CAPIOX FX05 membrane oxygenator group (MD = 48.96 beats/min, *p* < 0.001) and the ECLA Oxygenator group (MD = 50.58 beats/min, *p* < 0.001) exhibited significantly higher heart rates throughout the experimental period than the Conventional Oxygenator group. No statistically significant difference was observed between the two membrane oxygenator systems (MD = −1.63 beats/min, *p* > 0.999).

**Figure 7:**
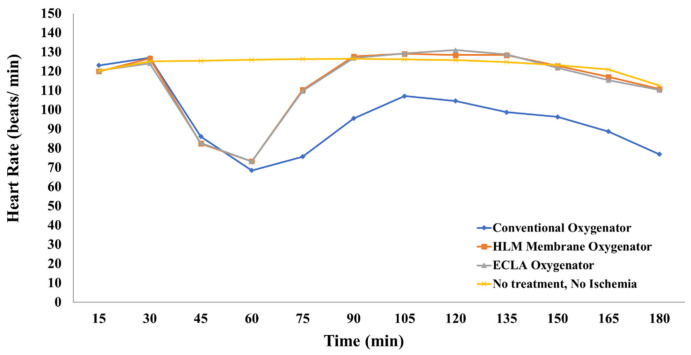
Changes in heart rate across groups over time.

The temporal evolution of heart rate is shown in Figure 7. Hearts perfused with membrane oxygenators demonstrated superior preservation of intrinsic cardiac rhythm during reperfusion compared with hearts perfused using the conventional bubble oxygenator. Comparable heart rate values between the HLM and ECLA groups further support the observation that both membrane oxygenation systems provided similar functional benefits.

#### 3.1.5. Rate Pressure Product (RPP)

The rate pressure product (RPP), calculated as the product of heart rate and left ventricular systolic pressure, is widely used as an integrated index of cardiac mechanical performance and myocardial work. Because it incorporates both chronotropic activity and systolic function, RPP is considered a sensitive indicator of overall myocardial recovery following ischemia–reperfusion injury [31,32].

The longitudinal analysis demonstrated significantly higher RPP values in hearts perfused using membrane oxygenation compared with the conventional bubble oxygenator. Both the CAPIOX FX05 membrane oxygenator group (MD = 9.51, *p* < 0.001) and the ECLA Oxygenator group (MD = 9.63, *p* < 0.001) exhibited significantly greater RPP throughout the experimental period than the Conventional Oxygenator group. No statistically significant difference was observed between the two membrane oxygenator systems (MD = −0.12, *p* > 0.999).

**Figure 8:**
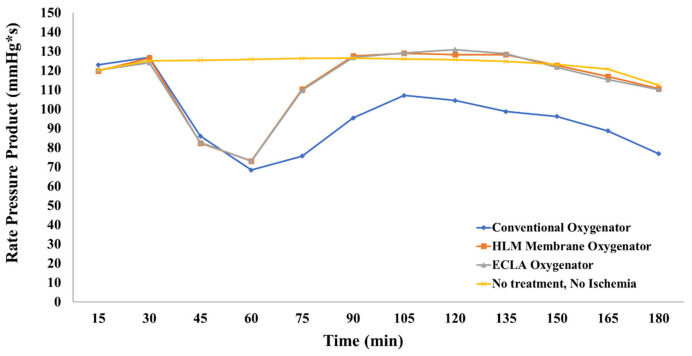
Changes in RPP across groups over time.

The temporal evolution of RPP is presented in Figure 8. Hearts perfused with membrane oxygenators demonstrated markedly improved overall cardiac performance during reperfusion compared with hearts perfused using the conventional bubble oxygenator. The similar RPP values observed in the HLM and ECLA groups further indicate that the beneficial effects were attributable to membrane oxygenation itself rather than to the specific oxygenator used.

#### 3.1.6. Maximum Positive First Derivative of Pressure (+dP/dt)

The maximum positive first derivative of left ventricular pressure (+dP/dt) is a widely accepted index of myocardial contractility and reflects the rate at which the ventricle develops pressure during systole. Higher +dP/dt values indicate improved contractile performance and enhanced recovery of myocardial function following ischemia–reperfusion injury [22,33].

The longitudinal analysis demonstrated significantly higher +dP/dt values in hearts perfused using membrane oxygenation compared with the conventional bubble oxygenator. Both the CAPIOX FX05 membrane oxygenator group (MD = 518.87 mmHg/s, *p* = 0.001) and the ECLA Oxygenator group (MD = 502.93 mmHg/s, *p* = 0.002) exhibited significantly greater +dP/dt throughout the experimental period than the Conventional Oxygenator group. No statistically significant difference was observed between the two membrane oxygenator systems (MD = 15.94 mmHg/s, *p* > 0.999).

**Figure 9:**
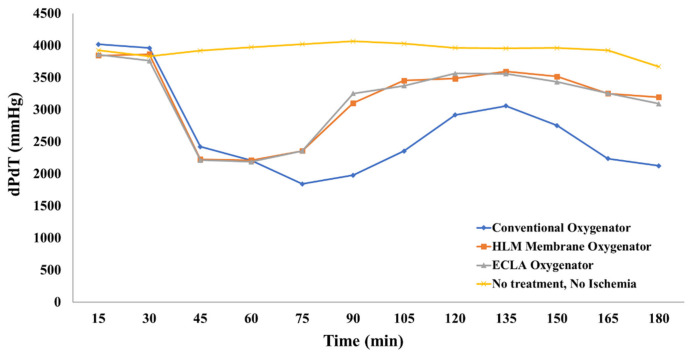
Changes in +dP/dt across groups over time.

The temporal evolution of +dP/dt is presented in Figure 9. Hearts perfused with membrane oxygenators demonstrated superior preservation of contractile function during reperfusion, as evidenced by the significantly higher rates of left ventricular pressure development compared with hearts perfused using the conventional bubble oxygenator. Similar +dP/dt values in the HLM and ECLA groups further indicate comparable efficacy of the two membrane oxygenation systems.

#### 3.1.7. Maximum Negative First Derivative of Pressure (−dP/dt)

The maximum negative first derivative of left ventricular pressure (−dP/dt) is a widely used index of myocardial relaxation and reflects the rate at which left ventricular pressure declines during diastole. Greater absolute values of −dP/dt indicate improved ventricular relaxation and better preservation of diastolic function following ischemia–reperfusion injury [22,33].

The longitudinal analysis demonstrated significantly improved −dP/dt values in hearts perfused using membrane oxygenation compared with the conventional bubble oxygenator. Both the CAPIOX FX05 membrane oxygenator group (MD = 357.16 mmHg/s, *p* < 0.001) and the ECLA Oxygenator group (MD = 361.44 mmHg/s, *p* < 0.001) exhibited significantly greater rates of left ventricular pressure decline throughout the experimental period than the Conventional Oxygenator group. No statistically significant difference was observed between the two membrane oxygenator systems (MD = −4.28 mmHg/s, *p* > 0.999).

**Figure 10:**
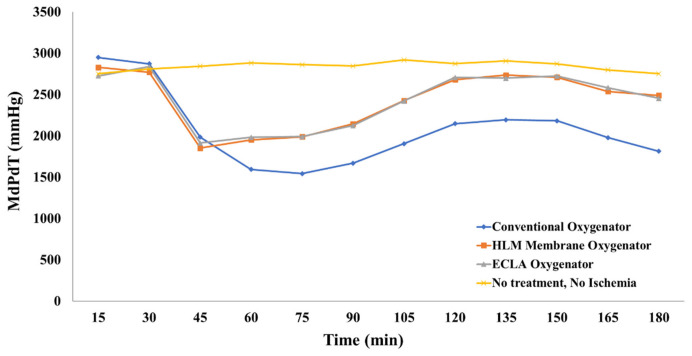
Changes in −dP/dt across groups over time.

The temporal evolution of −dP/dt is presented in Figure 10. Hearts perfused with membrane oxygenators demonstrated superior preservation of diastolic relaxation during reperfusion compared with hearts perfused using the conventional bubble oxygenator. Similar −dP/dt values in the HLM and ECLA groups further support the comparable efficacy of the two membrane oxygenation systems.

#### 3.1.8. Coronary Flow (CF)

Coronary flow (CF) reflects the adequacy of myocardial perfusion and provides an indirect assessment of coronary vascular function during isolated heart perfusion. Preservation of coronary flow following ischemia–reperfusion injury is generally associated with improved myocardial viability, enhanced microvascular integrity, and superior functional recovery [1,2,3].

The longitudinal analysis demonstrated significantly higher coronary flow values in hearts perfused using membrane oxygenation compared with the conventional bubble oxygenator. Both the CAPIOX FX05 membrane oxygenator group (MD = 6.32 mL/min, *p* < 0.001) and the ECLA Oxygenator group (MD = 6.50 mL/min, *p* < 0.001) exhibited significantly greater coronary flow throughout the experimental period than the Conventional Oxygenator group. No statistically significant difference was observed between the two membrane oxygenator systems (MD = −0.18 mL/min, *p* > 0.999).

**Figure 11:**
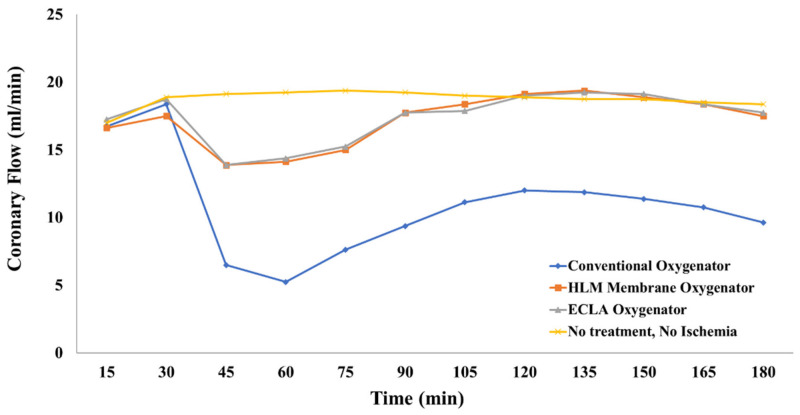
Changes in Coronary flow across groups over time.

The temporal evolution of coronary flow is presented in Figure 11. Hearts perfused with membrane oxygenators demonstrated significantly improved myocardial perfusion during reperfusion compared with hearts perfused using the conventional bubble oxygenator. The absence of significant differences between the HLM and ECLA groups further indicates that the observed improvement was attributable to membrane oxygenation rather than to characteristics of a specific oxygenator device.

#### 3.1.9. Perfusate pH

Perfusate pH was monitored throughout the experiment as an indicator of acid–base homeostasis and oxygenation stability. Maintenance of physiological pH is essential for normal myocardial function, and significant differences in pH between groups could potentially influence the interpretation of the observed hemodynamic findings [2,3,6].

The longitudinal analysis demonstrated no significant differences in perfusate pH between the membrane oxygenator groups and the Conventional Oxygenator group. The CAPIOX FX05 membrane oxygenator group (MD = 0.05, *p* = 0.475) and the ECLA Oxygenator group (MD = 0.04, *p* > 0.999) exhibited pH values comparable to those observed in the Conventional Oxygenator group. Similarly, no statistically significant difference was identified between the two membrane oxygenator systems (MD = 0.01, *p* > 0.999).

**Figure 12:**
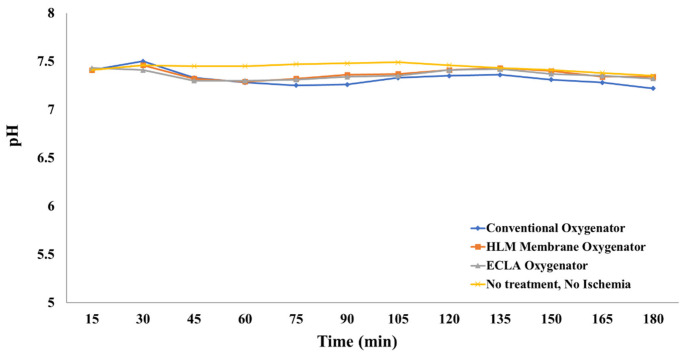
Changes in pH across groups over time.

The temporal evolution of perfusate pH is presented in Figure 12. All oxygenation systems maintained stable acid–base conditions throughout the experimental period. The absence of significant differences in pH among the experimental groups suggests that the improvements observed in myocardial performance and coronary flow following membrane oxygenation cannot be attributed to alterations in perfusate acid–base status.

#### 3.1.10. Carbozen Consumption

**Figure 13:**
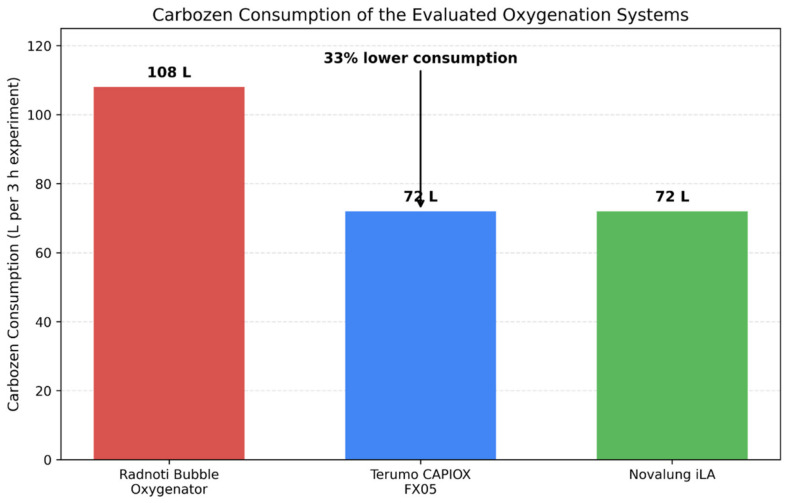
Carbozen gas consumption of the evaluated oxygenation systems. Adequate perfusate oxygenation was achieved with a gas flow of 0.4 L/min when membrane oxygenators were used, corresponding to a total Carbozen consumption of 72 L during a 3 h experiment. In contrast, the conventional bubble oxygenator required a gas flow of 0.6 L/min, resulting in a total consumption of 108 L. Membrane oxygenation therefore reduced Carbozen consumption by approximately 33%.

The use of membrane oxygenation was also associated with a substantial reduction in Carbozen gas consumption compared with the conventional bubble oxygenator. Adequate oxygenation of the perfusate was achieved with a gas flow of 0.4 L/min when either membrane oxygenator was used, corresponding to a total Carbozen consumption of approximately 72 L during a 3 h experiment. In contrast, operation of the conventional bubble oxygenator required a gas flow of 0.6 L/min, resulting in a total gas consumption of approximately 108 L over the same experimental period (Table 5, Figure 13).

Consequently, membrane oxygenation reduced Carbozen consumption by approximately 33% while maintaining comparable perfusate pO_2_, pCO_2_ and pH values and providing superior myocardial functional preservation. Based on a standard 10 L compressed Carbozen cylinder at 200 bar (approximately 2000 L gas capacity), this reduction would allow approximately 27 Langendorff experiments to be performed per cylinder using membrane oxygenation compared with approximately 18 experiments using the conventional bubble oxygenator. This reduction in gas consumption may allow a greater number of experiments to be performed using a standard gas cylinder. This finding represents an additional practical advantage of membrane oxygenation in isolated heart perfusion experiments providing a significant reduction in waste and cost.

The reduced gas flow requirements observed with membrane oxygenation were established during preliminary system optimization using intermittent blood gas measurements to confirm adequate perfusate oxygenation. However, because these measurements were not systematically collected throughout the experimental series, the present study cannot quantitatively compare oxygen transfer efficiency between the different oxygenation systems.

#### 3.1.11. Summary of the Oxygenator Comparison

Membrane oxygenation was associated with significantly improved preservation of myocardial function compared with the conventional bubble oxygenator across multiple hemodynamic parameters and coronary flow. In contrast, perfusate pH remained comparable among groups. No statistically significant differences were observed between the CAPIOX FX05 and Novalung iLA membrane oxygenators for any of the evaluated parameters.

### 3.2. Effect of Protein-Enriched Perfusate During Membrane Oxygenation

Following demonstration of the superiority of membrane oxygenation, a second experimental series was performed to evaluate whether supplementation of the perfusate with bovine serum albumin could further improve myocardial preservation during ischemia–reperfusion. The functional effects of protein-enriched perfusion were assessed by comparing standard Krebs–Henseleit buffer with a protein-supplemented perfusate under otherwise identical experimental conditions.

#### 3.2.1. Left Ventricular Systolic Pressure (LVSP)

The addition of bovine serum albumin to the perfusate resulted in a significant improvement in LVSP compared with standard KHB. Hearts perfused with the KHB + Protein Solution exhibited significantly higher LVSP values throughout the experimental period than hearts perfused with conventional KHB alone (MD = 4.62 mmHg, *p* = 0.044).

**Figure 14:**
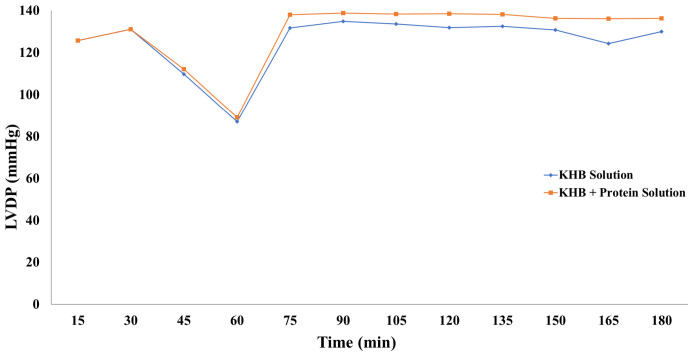
Changes in LVSP between KHB solution and KHB & Protein solution over time.

The temporal evolution of LVSP is presented in Figure 14. Although the magnitude of the improvement was less pronounced than that observed following the transition from bubble oxygenation to membrane oxygenation, protein supplementation provided a further enhancement of systolic ventricular performance. These findings suggest that optimization of perfusate composition may confer additional functional benefits beyond those achieved by improved oxygenation alone.

#### 3.2.2. Left Ventricular End-Diastolic Pressure (LVEDP)

Protein supplementation of the perfusate resulted in a significant reduction in LVEDP compared with standard KHB. Hearts perfused with the KHB + Protein Solution exhibited significantly lower LVEDP values throughout the experimental period than hearts perfused with KHB alone (MD = −2.29 mmHg, *p* < 0.001).

**Figure 15:**
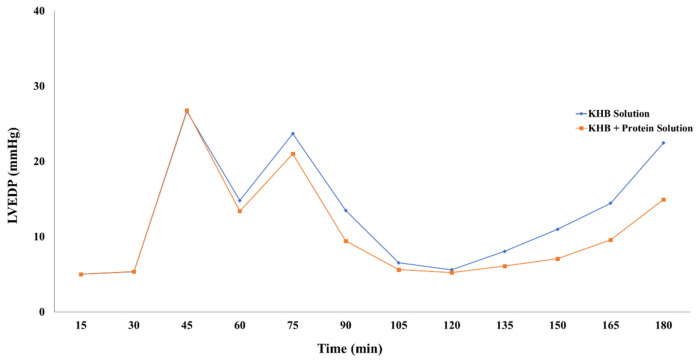
Changes in LVEDP between KHB solution and KHB & Protein solution over time.

The temporal evolution of LVEDP is presented in Figure 15. The observed reduction in LVEDP indicates improved preservation of diastolic ventricular function during reperfusion in the protein-supplemented group. Notably, the effect on LVEDP was proportionally more pronounced than the improvement observed for LVSP, suggesting that protein supplementation may have exerted a particularly beneficial influence on ventricular compliance and diastolic performance.

#### 3.2.3. Left Ventricular Developed Pressure (LVDP)

Protein supplementation of the perfusate resulted in a significant improvement in LDP compared with standard KHB. Hearts perfused with the KHB + Protein Solution exhibited significantly higher LVDP values throughout the experimental period than hearts perfused with KHB alone (MD = 6.91 mmHg, *p* = 0.010).

**Figure 16:**
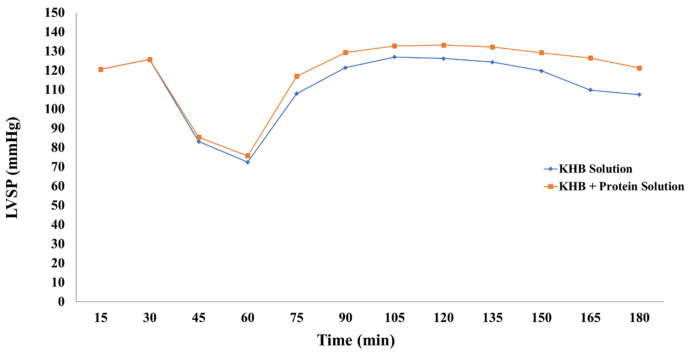
Changes in LVDP between KHB solution and KHB & Protein solution over time.

The temporal evolution of LVDP is presented in Figure 16. The observed increase in LVDP indicates superior preservation of overall ventricular function in the protein-supplemented group during reperfusion. Notably, the magnitude of improvement in LVDP exceeded that observed for LVSP alone, suggesting that the beneficial effects of protein supplementation were attributable to improvements in both systolic and diastolic myocardial performance. These findings further support the concept that optimization of perfusate composition can enhance functional recovery beyond that achieved by membrane oxygenation alone.

#### 3.2.4. Heart Rate (HR)

No statistically significant difference in HR was observed between the KHB + Protein Solution group and the KHB Solution group (MD = 19.47 beats/min, *p* = 0.055), although a trend towards higher heart rates was noted in the protein-supplemented group.

**Figure 17:**
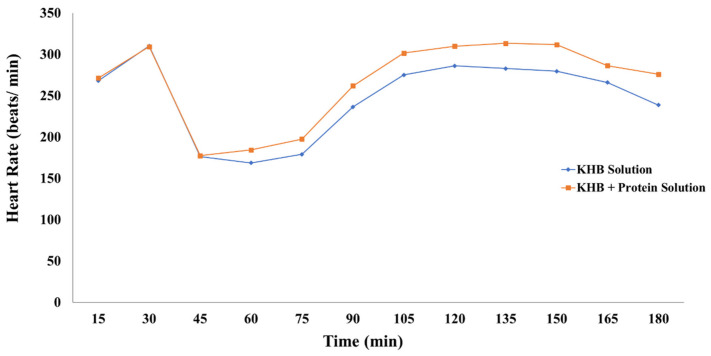
Changes in heart rate between KHB solution and KHB & Protein solution over time.

In contrast to the improvements observed in several indices of ventricular performance, protein supplementation did not significantly affect the intrinsic chronotropic activity of the isolated hearts. These findings suggest that the beneficial effects of protein supplementation were primarily related to preservation of myocardial mechanical function rather than alterations in cardiac rhythm generation (Figure 17).

#### 3.2.5. Rate Pressure Product (RPP)

Protein supplementation of the perfusate resulted in a significant increase in RPP compared with standard KHB. Hearts perfused with the KHB + Protein Solution exhibited significantly higher RPP values throughout the experimental period than hearts perfused with KHB alone (MD = 4.14, *p* = 0.013).

**Figure 18:**
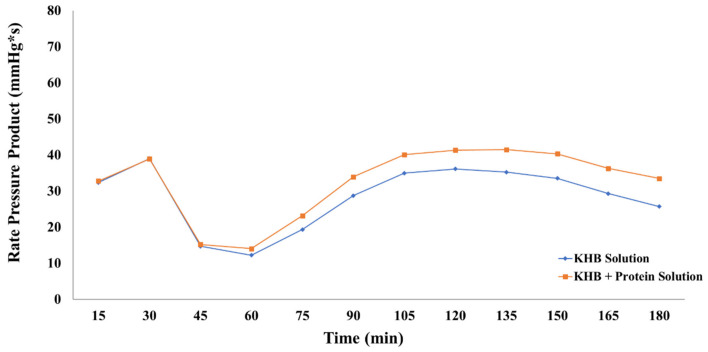
Changes in RPP between KHB solution and KHB & Protein solution over time.

The temporal evolution of RPP is presented in Figure 18. The observed increase in RPP indicates enhanced overall cardiac mechanical performance in the protein-supplemented group during reperfusion. Notably, despite the absence of a statistically significant effect on heart rate, protein supplementation significantly improved RPP, suggesting that the observed benefit was primarily driven by enhanced ventricular function rather than changes in chronotropic activity. These findings further support the concept that optimization of perfusate composition contributes to improved functional recovery following ischemia–reperfusion injury.

#### 3.2.6. Maximum Positive First Derivative of Pressure (+dP/dt)

**Figure 19:**
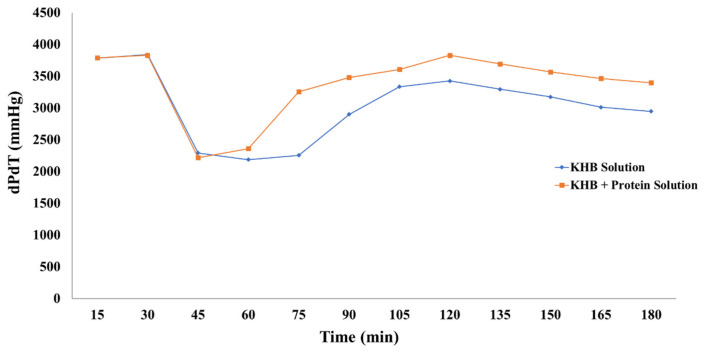
Changes in +dP/dt between KHB solution and KHB & Protein solution over time.

No statistically significant difference in +dP/dt was observed between the KHB + Protein Solution group and the KHB Solution group (MD = 337.58 mmHg/s, *p* = 0.092). Nevertheless, a numerical trend towards higher +dP/dt values was evident in hearts perfused with the protein-supplemented solution (Figure 19).

Although protein supplementation did not significantly enhance the rate of left ventricular pressure development, the observed trend suggests a potential improvement in myocardial contractile performance. In contrast to the significant effects observed for LVSP, LVEDP, LVDP, and RPP, the lack of statistical significance for +dP/dt indicates that the beneficial effects of protein supplementation may have been more pronounced on global ventricular performance than on the intrinsic contractile properties of the myocardium.

#### 3.2.7. Maximum Negative First Derivative of Pressure (−dP/dt)

Protein supplementation of the perfusate resulted in a significant improvement in −dP/dt compared with standard Krebs–Henseleit buffer. Hearts perfused with the KHB + Protein Solution exhibited significantly greater rates of left ventricular pressure decline during diastole than hearts perfused with KHB alone (MD = 315.31 mmHg/s, *p* < 0.001).

**Figure 20:**
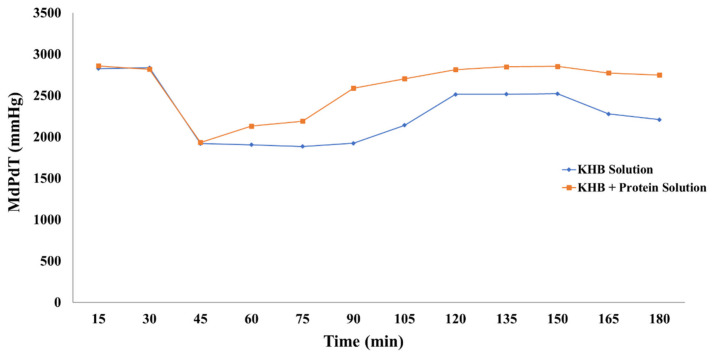
Changes in −dP/dt between KHB solution and KHB & Protein solution over time.

The observed increase in the magnitude of −dP/dt indicates enhanced ventricular relaxation and improved preservation of diastolic function in the protein-supplemented group during reperfusion. Notably, whereas the effect of protein supplementation on +dP/dt did not reach statistical significance, a significant improvement was observed for −dP/dt, suggesting that the beneficial effects of protein supplementation may have been particularly pronounced on myocardial relaxation and ventricular compliance. These findings are consistent with the overall pattern observed for LVEDP and support a favorable effect of protein-enriched perfusion on diastolic myocardial performance (Figure 20).

#### 3.2.8. Coronary Flow (CF)

Protein supplementation of the perfusate resulted in a significant increase in CF compared with standard KHB. Hearts perfused with the KHB + Protein Solution exhibited significantly higher CF throughout the experimental period than hearts perfused with KHB alone (MD = 5.57 mL/min, *p* < 0.001).

**Figure 21:**
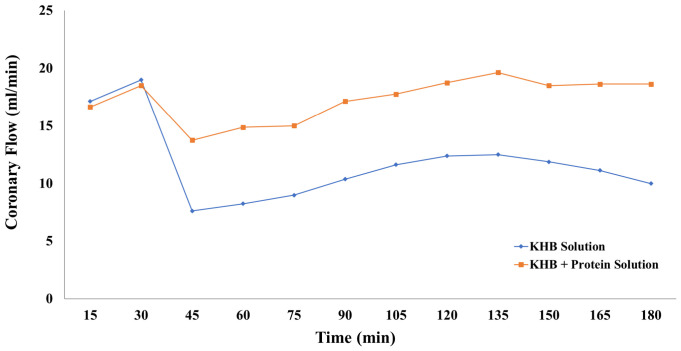
Changes in Coronary flow between KHB solution and KHB & Protein solution over time.

The temporal evolution of CF is presented in Figure 21. The observed increase in coronary flow indicates improved myocardial perfusion in the protein-supplemented group during reperfusion. Notably, the magnitude and statistical significance of the observed effect were among the most pronounced findings of the protein supplementation experiment. Together with the improvements observed in LVEDP, LVDP, RPP, and −dP/dt, these findings suggest that protein-enriched perfusion promoted superior preservation of both myocardial function and coronary vascular performance following ischemia–reperfusion injury.

#### 3.2.9. Perfusate pH

**Figure 22:**
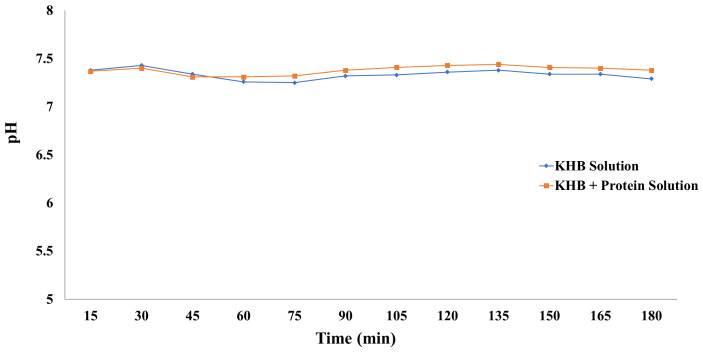
Changes in pH between KHB solution and KHB & Protein solution over time.

No statistically significant difference in perfusate pH was observed between the KHB + Protein Solution group and the KHB Solution group (MD = 0.05, *p* = 0.475). Both perfusion solutions maintained stable acid–base conditions throughout the experimental period, and the addition of bovine serum albumin did not significantly affect perfusate pH. Consequently, the improvements observed in myocardial function and coronary flow in the protein-supplemented group cannot be attributed to alterations in acid–base homeostasis but are more likely related to the physiological effects of protein enrichment of the perfusate (Figure 22).

#### 3.2.10. Summary of the Protein Supplementation Experiment

Taken together, the results demonstrate that supplementation of the perfusate with bovine serum albumin provided additional functional benefits beyond those achieved by membrane oxygenation alone. Compared with standard Krebs–Henseleit buffer, protein-enriched perfusion resulted in significantly higher LVSP, LVDP, RPP, −dP/dt, and coronary flow, together with significantly lower LVEDP. In contrast, no significant differences were observed for heart rate, +dP/dt, or perfusate pH.

The overall pattern of results suggests that protein supplementation primarily enhanced preservation of ventricular function and myocardial perfusion during reperfusion. The particularly pronounced effects observed for LVEDP, −dP/dt, and coronary flow indicate a beneficial influence on diastolic performance and coronary vascular function. These findings demonstrate that the incorporation of protein into the perfusate, made possible through the use of membrane oxygenation, represents an additional strategy for optimizing the Langendorff isolated heart model.

## 4. Discussion

### 4.1. Principal Findings

The present study was designed to evaluate whether replacement of the conventional bubble oxygenator with membrane oxygenation could improve the performance of the Langendorff isolated heart model and facilitate the use of physiologically enhanced perfusion solutions. The principal findings can be summarized as follows. First, both membrane oxygenators tested, namely the CAPIOX^®^ FX05 and the Novalung iLA^®^, provided significantly superior myocardial functional preservation compared with the conventional bubble oxygenator, as demonstrated by improvements in LVSP, LVEDP, LVDP, RPP, ±dP/dt, and coronary flow. Second, no significant differences were observed between the two membrane oxygenators, suggesting that the observed benefits were attributable to membrane oxygenation itself rather than to a specific device design. Third, membrane oxygenation enabled stable supplementation of the perfusate with bovine serum albumin, a modification that is impractical in conventional bubble oxygenation systems due to excessive foam formation. Finally, protein enrichment of the perfusate provided additional improvements in myocardial performance and coronary perfusion, while membrane oxygenation simultaneously reduced Carbozen consumption by approximately one third.

The Langendorff isolated heart preparation remains one of the most widely used experimental platforms for investigating cardiac physiology, ischemia–reperfusion injury, pharmacological interventions, and myocardial protection strategies [1,2,3,4,5,6,34,35,36,37]. Despite its widespread adoption, the basic principles of the model have remained largely unchanged for more than a century, and several limitations of conventional crystalloid perfusion systems continue to persist [3,34,38,39]. Among these limitations are the absence of physiological colloid osmotic pressure, progressive myocardial fluid accumulation during prolonged perfusion, and the restricted ability to modify perfusate composition when direct gas bubbling is used for oxygenation [7,8,9,10,38]. Experimental and clinical studies have demonstrated that myocardial edema adversely affects ventricular compliance, myocardial contractility, coronary microvascular function, and overall cardiac performance, emphasizing the importance of maintaining myocardial fluid homeostasis during ex vivo perfusion [7,8,9,10,40,41]. The findings of the present study suggest that membrane oxygenation may address several of these limitations simultaneously and therefore represents a potentially important refinement of the conventional Langendorff methodology.

The principal findings of the present study are the observed improvements in myocardial performance and coronary flow associated with membrane oxygenation. Specifically, membrane oxygenation was associated with higher LVSP, LVDP, heart rate, rate-pressure product, ±dP/dt, and coronary flow, together with lower LVEDP compared with conventional bubble oxygenation. The mechanisms underlying these improvements were not directly investigated. Therefore, potential explanations, including more efficient oxygen transfer, reduced gaseous microembolization, improved microvascular perfusion, or enhanced myocardial oxygen delivery, should be regarded as plausible hypotheses rather than experimentally proven mechanisms.

Importantly, the benefits observed in the present investigation extended beyond simple improvements in oxygen delivery. Although enhanced gas exchange likely contributed to the superior functional preservation observed in the membrane oxygenator groups, the elimination of direct gas–liquid interaction also enabled stable use of a protein-enriched perfusate, thereby introducing an additional level of physiological optimization that could not be achieved with conventional bubble oxygenation. The combination of improved myocardial performance, enhanced coronary perfusion, preservation of acid–base stability, and reduced Carbozen consumption suggests that membrane oxygenation should not be regarded merely as an alternative oxygenation strategy but rather as an enabling technology that expands the experimental capabilities of isolated heart perfusion models [3,16,17,18,34,42]. The mechanistic basis of these observations and their implications for future isolated organ perfusion research are discussed in the following sections.

### 4.2. Mechanisms Underlying the Benefits of Membrane Oxygenation

The superiority of membrane oxygenation observed in the present study was reflected consistently across virtually all measured indicators of myocardial performance, including systolic function (LVSP, LVDP, +dP/dt), diastolic function (LVEDP, −dP/dt), myocardial work (RPP), and coronary perfusion. The fact that these improvements occurred simultaneously and were observed with two different membrane oxygenator systems strongly suggests that the beneficial effects were related to the mode of oxygenation itself rather than to device-specific characteristics. Although the present study was not designed to directly quantify oxygen transfer kinetics, several physiological mechanisms may explain the observed findings.

Membrane oxygenators have become the standard technology for gas exchange in modern cardiopulmonary bypass and extracorporeal life support because they provide efficient oxygen transfer while physically separating the gas and liquid phases through a semipermeable membrane [16,17,18]. In contrast, conventional bubble oxygenators rely on direct gas–liquid interaction, a process that may generate gas microbubbles, increase shear forces within the perfusate, and produce less controlled gas exchange conditions [43,44]. The elimination of direct gas bubbling may therefore contribute to a more stable perfusion environment and reduce disturbances within the coronary circulation. Although microbubble formation was not specifically assessed in the present study, previous investigations have demonstrated that gaseous microemboli may adversely affect microvascular perfusion and tissue oxygen delivery, even when present in relatively small quantities [3,43,44].

The significant improvements in coronary flow observed in both membrane oxygenator groups may provide indirect support for this concept. Adequate coronary perfusion is essential for myocardial oxygen delivery and maintenance of contractile function during reperfusion following ischemic injury. Experimental studies have shown that disturbances at the level of the coronary microcirculation may substantially impair post-ischemic functional recovery, independent of epicardial coronary patency [45,46]. In the present study, membrane oxygenation was associated with higher coronary flow throughout the reperfusion period, suggesting improved preservation of coronary vascular function. The concomitant improvements observed in LVSP, LVDP, RPP, and ±dP/dt are consistent with this interpretation and indicate that the benefits of membrane oxygenation extended beyond simple maintenance of acid–base balance.

An additional observation supporting a genuine physiological benefit of membrane oxygenation was the absence of clinically meaningful differences in perfusate pH among the experimental groups. Had the observed improvements been solely attributable to superior acid–base control, significant alterations in pH would have been expected. Instead, all oxygenation systems maintained comparable acid–base conditions throughout the experiment, while marked differences in myocardial performance remained evident. This finding suggests that membrane oxygenation exerted beneficial effects through mechanisms other than global acid–base regulation and may reflect improved oxygen delivery, enhanced preservation of the myocardial microenvironment, or superior maintenance of microvascular perfusion [16,17,18,47].

Interestingly, no significant differences were identified between the CAPIOX^®^ FX05 and the Novalung iLA^®^ oxygenators despite their different original clinical applications. The CAPIOX^®^ FX05 was originally developed for pediatric cardiopulmonary bypass, whereas the Novalung iLA^®^ was designed for extracorporeal lung assist applications. Nevertheless, both systems produced virtually identical results across all measured parameters. This observation further strengthens the conclusion that the observed benefits were attributable to the fundamental principle of membrane-mediated gas exchange rather than to a particular oxygenator design. From a practical perspective, these findings suggest that a variety of commercially available membrane oxygenators may be suitable for incorporation into Langendorff perfusion systems, allowing investigators to select devices according to local availability and economic considerations without compromising experimental performance.

The superior functional preservation observed with membrane oxygenation is consistent with more effective gas exchange and avoidance of the potential disadvantages associated with direct gas bubbling. However, because oxygen delivery, dissolved oxygen content, oxygen extraction, and myocardial oxygen consumption were not directly measured, the precise mechanisms responsible for these improvements cannot be definitively established. Consequently, the proposed explanations should be considered plausible interpretations of the observed functional findings rather than proven mechanistic conclusions.

These findings should also be interpreted in the context of the recognized oxygen transport limitations of crystalloid-perfused isolated heart preparations, as discussed in the Introduction. Previous studies have demonstrated that myocardial performance in isolated heart models may become constrained by oxygen delivery despite apparently adequate perfusion conditions, highlighting the potential importance of optimizing oxygen transfer within the perfusion circuit [11,12,13].

Similarly, the beneficial effects observed with bovine serum albumin supplementation may be related to improved maintenance of colloid osmotic pressure and fluid balance during prolonged perfusion. Nevertheless, myocardial edema was not directly assessed by wet-to-dry weight ratios, tissue water content measurements, or histological examination. Therefore, the proposed role of reduced edema formation remains speculative and warrants further investigation.

### 4.3. Membrane Oxygenation Enables Protein-Enriched Perfusion

Beyond the direct improvements in myocardial performance observed with membrane oxygenation, one of the most important findings of the present study was the ability to utilize a protein-enriched perfusate. Although the physiological advantages of colloid-containing perfusion solutions have been recognized for decades, their routine use in conventional Langendorff systems has remained limited because direct gas bubbling through protein-containing solutions results in excessive foam formation and unstable perfusion conditions [7,9,48]. In the present study, replacement of the conventional bubble oxygenator with membrane oxygenation completely eliminated this technical limitation and enabled stable supplementation of the perfusate with bovine serum albumin throughout the experimental period.

This observation has implications that extend beyond the specific experimental protocol employed in the present investigation. By physically separating the gas and liquid phases, membrane oxygenators allow greater flexibility in perfusate design and facilitate the incorporation of physiologically relevant constituents that would otherwise be difficult to use in conventional systems. Consequently, membrane oxygenation should not be viewed solely as a means of improving gas exchange but rather as a platform technology that enables further optimization of isolated organ perfusion models. The additional functional benefits observed following protein supplementation in the present study support this concept and suggest that future refinements of the Langendorff model may be achieved not only through improvements in oxygenation technology but also through the development of more physiologically representative perfusion solutions.

### 4.4. Functional Benefits of Protein Supplementation

Following establishment of membrane oxygenation as the preferred oxygenation strategy, supplementation of the perfusate with bovine serum albumin resulted in additional improvements in myocardial performance. As mentioned before, a concentration of 1% BSA was selected as a pragmatic and commonly used protein supplementation strategy intended to increase colloid osmotic pressure while maintaining favorable perfusion characteristics; optimization of albumin concentration was beyond the scope of the present investigation.

The most pronounced effects were observed for LVEDP, −dP/dt, LVDP, and coronary flow, suggesting that protein supplementation exerted a particularly beneficial influence on diastolic function and myocardial perfusion. Interestingly, the improvement in LVEDP and the enhanced rate of ventricular relaxation (−dP/dt) were proportionally greater than the corresponding changes in indices of systolic function, indicating that the protein-enriched perfusate may have primarily affected ventricular compliance and myocardial mechanical properties rather than intrinsic contractility alone.

A plausible explanation for these findings is the partial restoration of colloid osmotic pressure within the perfusion circuit. Conventional crystalloid-based Langendorff perfusion solutions lack plasma proteins and therefore do not reproduce the oncotic forces present under physiological conditions. As a consequence, fluid may progressively accumulate within the myocardial interstitium during prolonged perfusion, particularly following ischemia–reperfusion injury, where microvascular permeability is increased [8,40,41,49]. Previous experimental studies have demonstrated that myocardial edema is associated with impaired ventricular compliance, reduced coronary perfusion, and diminished functional recovery [7,9,37,50]. Although myocardial water content was not directly quantified in the present study, the combination of lower LVEDP, improved −dP/dt, and higher coronary flow observed following protein supplementation is consistent with improved myocardial fluid balance and preservation of diastolic function.

However, the present study demonstrates functional correlates consistent with reduced edema but does not directly establish edema reduction as the causal mechanism. Measurements such as myocardial wet-to-dry weight ratios, tissue water content analysis, or histological assessment of myocardial edema were not performed. Consequently, the proposed relationship between albumin supplementation, edema reduction, and improved myocardial function should be regarded as a hypothesis supported by physiological plausibility rather than a mechanism directly demonstrated by the present data.

The present findings are also in agreement with previous investigations demonstrating beneficial effects of albumin-containing perfusion solutions during isolated organ preservation and reperfusion [25,26,27,28,51,52,53]. Importantly, the current study extends these observations by demonstrating that such a strategy can be readily incorporated into a Langendorff preparation when membrane oxygenation is employed. Taken together, these results suggest that the physiological advantages of membrane oxygenation may arise not only from improved gas exchange but also from the opportunity to utilize more physiologically representative perfusion solutions that better preserve myocardial structure and function during prolonged ex vivo perfusion.

### 4.5. Practical and Economic Advantages of Membrane Oxygenation

In addition to the physiological benefits observed in the present study, membrane oxygenation provided a number of practical advantages that may facilitate routine implementation in experimental laboratories. Both membrane oxygenators achieved adequate perfusate oxygenation using a Carbozen flow rate of 0.4 L/min, whereas the conventional bubble oxygenator required 0.6 L/min. This translated into an approximately 33% reduction in gas consumption and increased the number of 3 h Langendorff experiments that could be performed using a standard 10 L compressed Carbozen cylinder from approximately 18 to 27. Such a reduction may improve the cost-effectiveness of prolonged experimental protocols and decrease the logistical burden associated with gas supply, particularly in laboratories performing large experimental series. Furthermore, the absence of direct gas bubbling simplified perfusate handling and eliminated foam formation during protein supplementation, thereby improving overall system stability.

Although not a primary endpoint of the study, the reduced gas requirements associated with membrane oxygenation may provide practical advantages for laboratories performing large numbers of Langendorff experiments. Beyond reducing consumable usage, lower gas requirements may improve logistical efficiency and decrease the frequency of gas cylinder replacement. These practical considerations are secondary to the physiological findings of the present study but may facilitate implementation of membrane oxygenation in routine experimental workflows. Collectively, these practical advantages complement the physiological benefits of membrane oxygenation and further support its use as a refinement of the conventional Langendorff perfusion model.

## 5. Future Perspectives

The findings of the present study open several avenues for further refinement of the Langendorff isolated heart model. Future investigations should focus on direct assessment of myocardial edema through determination of tissue water content, wet-to-dry weight ratios, and histological analysis in order to better characterize the mechanisms underlying the observed benefits of protein-enriched perfusion. In addition, direct measurements of oxygen transfer efficiency, dissolved oxygen content, and myocardial oxygen consumption may provide further insight into the physiological advantages of membrane oxygenation compared with conventional bubble oxygenation.

Beyond the specific experimental protocol employed in the present study, membrane oxygenation may facilitate the development of more physiologically representative perfusion solutions containing proteins, metabolic substrates, pharmacological carriers, or blood-derived components that are difficult to use in conventional bubble oxygenation systems. Such modifications could further improve myocardial preservation during prolonged ex vivo perfusion and increase the translational relevance of isolated organ experiments. Furthermore, the application of membrane oxygenation to extended-duration perfusion protocols, large-animal models, and ex vivo organ preservation systems warrants investigation.

Beyond the findings reported in the present study, membrane oxygenation may facilitate the use of more physiologically relevant perfusion solutions that are difficult or impossible to employ in conventional bubble-oxygenated systems. Potential future applications include erythrocyte-containing perfusates, plasma-based solutions, artificial oxygen carriers such as perfluorocarbons, and metabolically enriched perfusion media incorporating proteins, hormones, and other biologically active components. Such approaches may improve oxygen transport capacity and better reproduce the physiological environment of the myocardium during ex vivo perfusion. In this context, membrane oxygenation may represent an enabling platform for the next generation of Langendorff perfusion models and a further step toward more physiological ex vivo cardiac experimentation.

Collectively, these future developments may contribute to the establishment of a new generation of isolated organ perfusion platforms that more closely reproduce physiological conditions while maintaining the experimental flexibility that has made the Langendorff model an indispensable tool in cardiovascular research.

## 6. Limitations

Several limitations of the present study should be acknowledged. First, although the use of a protein-enriched perfusate was associated with significant improvements in ventricular function and coronary flow, direct quantification of myocardial edema was not performed. Consequently, parameters such as myocardial water content, wet-to-dry weight ratio, or histological assessment of tissue fluid accumulation were not available to confirm the precise mechanisms responsible for the observed functional benefits. While the findings are consistent with improved myocardial fluid balance, a direct causal relationship cannot be established on the basis of the present data alone.

Second, the physiological advantages of membrane oxygenation were inferred from functional and hemodynamic outcomes rather than from direct measurements of oxygen transfer efficiency. Variables such as perfusate oxygen tension, oxygen extraction, and myocardial oxygen consumption were not assessed, and therefore the exact contribution of enhanced oxygen delivery remains to be determined.

An important limitation of the present study is that the mechanisms underlying the observed functional improvements were not directly investigated. Although membrane oxygenation was associated with superior myocardial performance and coronary flow, measurements of perfusate oxygen tension, dissolved oxygen content, oxygen extraction, or myocardial oxygen consumption were not performed. Therefore, the observed benefits should be considered consistent with, but not definitive proof of, improved oxygen delivery. Similarly, while supplementation with bovine serum albumin was associated with improved functional preservation, myocardial edema was not directly quantified using wet-to-dry weight ratios, tissue water content measurements, or histological assessment. Consequently, the proposed role of improved colloid osmotic support and reduced edema formation remains speculative and requires confirmation in future studies specifically designed to evaluate these mechanisms.

Although perfusate pO_2_ and oxygen saturation measurements were used during preliminary system standardization to establish appropriate gas flow settings for each oxygenation system, these variables were not systematically recorded throughout all experiments and were therefore not available for formal statistical analysis. Consequently, the present study cannot directly compare oxygen transfer efficiency between the different oxygenation systems. Future studies incorporating serial measurements of perfusate oxygen tension, oxygen saturation, dissolved oxygen content, and myocardial oxygen consumption would provide valuable mechanistic insight into the functional differences observed between bubble and membrane oxygenation.

An additional limitation is that the two experimental series were performed sequentially rather than simultaneously. Although identical experimental protocols, equipment, laboratory conditions, and investigators were used throughout the study, the possibility of unrecognized batch effects cannot be completely excluded. To minimize this risk, independent animal cohorts were used for each experimental series, and comparisons within the second series were performed using contemporaneous control and intervention groups studied under identical conditions.

Finally, the study was performed in an isolated rat heart model using a standardized protocol of global regional ischemia–reperfusion injury. Although the Langendorff preparation represents a well-established and highly reproducible experimental platform, extrapolation of the present findings to other species, longer perfusion durations, or alternative organ perfusion systems should be undertaken with caution. Nevertheless, the consistent improvements observed across multiple independent indices of myocardial performance support the robustness of the overall conclusions and provide a strong rationale for further investigation of membrane oxygenation-based perfusion strategies.

## 7. Conclusions

The present study demonstrates that membrane oxygenation represents a significant refinement of the Langendorff isolated heart model. Compared with conventional bubble oxygenation, membrane oxygenators provided superior preservation of myocardial function and coronary perfusion while simultaneously reducing Carbozen consumption. Furthermore, membrane oxygenation enabled stable protein supplementation of the perfusate, resulting in additional improvements in cardiac performance. Taken together, these findings suggest that membrane oxygenation not only improves oxygen delivery but also facilitates the development of more physiologically relevant perfusion strategies, thereby enhancing the experimental and translational value of the Langendorff model.

## Figures and Tables

**Table 1:** Pairwise comparisons for all measured parameters among the experimental groups.

		Mean Difference	*p*
LVSP			
HLM Membrane Oxygenator	Conventional Oxygenator	13.78	<0.001
ECLA Oxygenator	Conventional Oxygenator	13.75	<0.001
No treatment, No Ischemia	Conventional Oxygenator	16.65	<0.001
HLM Membrane Oxygenator	ECLA Oxygenator	0.03	<0.999
KHB + Protein Solution	KHB Solution	4.62	0.044
LVEDP			
HLM Membrane Oxygenator	Conventional Oxygenator	−5.27	<0.001
ECLA Oxygenator	Conventional Oxygenator	−5.02	<0.001
No treatment, No Ischemia	Conventional Oxygenator	−11.25	<0.001
HLM Membrane Oxygenator	ECLA Oxygenator	−0.26	<0.999
KHB + Protein Solution	KHB Solution	−2.29	<0.001
LVDP			
HLM Membrane Oxygenator	Conventional Oxygenator	19.05	<0.001
ECLA Oxygenator	Conventional Oxygenator	18.77	<0.001
No treatment, No Ischemia	Conventional Oxygenator	27.91	<0.001
HLM Membrane Oxygenator	ECLA Oxygenator	0.28	<0.999
KHB + Protein Solution	KHB Solution	6.91	0.010
HR			
HLM Membrane Oxygenator	Conventional Oxygenator	48.96	<0.001
ECLA Oxygenator	Conventional Oxygenator	50.58	<0.001
No treatment, No Ischemia	Conventional Oxygenator	94.96	<0.001
HLM Membrane Oxygenator	ECLA Oxygenator	−1.63	<0.999
KHB + Protein Solution	KHB Solution	19.47	0.055
RPR			
HLM Membrane Oxygenator	Conventional Oxygenator	9.51	<0.001
ECLA Oxygenator	Conventional Oxygenator	9.63	<0.001
No treatment, No Ischemia	Conventional Oxygenator	16.75	<0.001
HLM Membrane Oxygenator	ECLA Oxygenator	−0.12	<0.999
KHB + Protein Solution	KHB Solution	4.14	0.013
dPdT			
HLM Membrane Oxygenator	Conventional Oxygenator	518.87	0.001
ECLA Oxygenator	Conventional Oxygenator	502.93	0.002
No treatment, No Ischemia	Conventional Oxygenator	1280.42	<0.001
HLM Membrane Oxygenator	ECLA Oxygenator	15.94	<0.999
KHB + Protein Solution	KHB Solution	337.58	0.092
MdPdT			
HLM Membrane Oxygenator	Conventional Oxygenator	357.16	<0.001
ECLA Oxygenator	Conventional Oxygenator	361.44	<0.001
No treatment, No Ischemia	Conventional Oxygenator	773.30	<0.001
HLM Membrane Oxygenator	ECLA Oxygenator	−4.28	<0.999
KHB + Protein Solution	KHB Solution	315.31	<0.001
CF			
HLM Membrane Oxygenator	Conventional Oxygenator	6.32	<0.001
ECLA Oxygenator	Conventional Oxygenator	6.50	<0.001
No treatment, No Ischemia	Conventional Oxygenator	7.87	<0.001
HLM Membrane Oxygenator	ECLA Oxygenator	−0.18	<0.999
KHB + Protein Solution	KHB Solution	5.57	<0.001
pH			
HLM Membrane Oxygenator	Conventional Oxygenator	0.05	0.475
ECLA Oxygenator	Conventional Oxygenator	0.04	<0.999
No treatment, No Ischemia	Conventional Oxygenator	0.11	<0.001
HLM Membrane Oxygenator	ECLA Oxygenator	0.01	<0.999
KHB + Protein Solution	KHB Solution	0.05	0.475

**Table 2:** Longitudinal analysis for all measurements for CAPIOX FX05 (HLM) membrane oxygenator, ECLA Oxygenator versus Conventional Oxygenator.

LVSP	b	*p*	95% CI
Time	0.442	0.012	0.099, 0.786
Group (Conventional Oxygenator: Ref. category)			
HLM Membrane Oxygenator	13.776	<0.001	10.424, 17.129
ECLA Oxygenator	13.748	<0.001	10.396, 17.101
No treatment, No Ischemia	16.654	<0.001	13.301, 20.006
LVEDP			
Time	0.288	0.007	0.079, 0.496
Group (Conventional Oxygenator: Ref. category)			
HLM Membrane Oxygenator	−5.273	<0.001	−7.306, −3.24
ECLA Oxygenator	−5.018	<0.001	−7.051, −2.985
No treatment, No Ischemia	−11.256	<0.001	−13.288, −9.223
LVDP			
Time	0.155	0.512	−0.308, 0.618
Group (Conventional Oxygenator: Ref. category)			
HLM Membrane Oxygenator	19.049	<0.001	14.528, 23.57
ECLA Oxygenator	18.766	<0.001	14.245, 23.287
No treatment, No Ischemia	27.909	<0.001	23.388, 32.43
HR			
Time	0.516	0.439	−0.792, 1.824
Group (Conventional Oxygenator: Ref. category)			
HLM Membrane Oxygenator	48.958	<0.001	36.188, 61.728
ECLA Oxygenator	50.583	<0.001	37.813, 63.353
No treatment, No Ischemia	94.958	<0.001	82.188, 107.728
RPR			
Time	0.031	0.798	−0.207, 0.269
Group (Conventional Oxygenator: Ref. category)			
HLM Membrane Oxygenator	9.506	<0.001	7.183, 11.828
ECLA Oxygenator	9.626	<0.001	7.304, 11.949
No treatment, No Ischemia	16.746	<0.001	14.424, 19.069
dPdT			
Time	−13.071	0.114	−29.283, 3.141
Group (Conventional Oxygenator: Ref. category)			
HLM Membrane Oxygenator	518.865	<0.001	283.628, 754.101
ECLA Oxygenator	502.927	<0.001	267.691, 738.163
No treatment, No Ischemia	1280.417	<0.001	1045.18, 1515.653
MdPdT			
Time	−0.746	0.881	−10.518, 9.026
Group (Conventional Oxygenator: Ref. category)			
HLM Membrane Oxygenator	357.156	<0.001	246.039, 468.274
ECLA Oxygenator	361.438	<0.001	250.32, 472.555
No treatment, No Ischemia	773.302	<0.001	662.185, 884.42
CF			
Time	0.093	0.01	0.023, 0.163
Group (Conventional Oxygenator: Ref. category)			
HLM Membrane Oxygenator	6.323	<0.001	4.867, 7.779
ECLA Oxygenator	6.5	<0.001	5.044, 7.956
No treatment, No Ischemia	7.875	<0.001	6.419, 9.331
pH			
Time	−0.005	<0.001	−0.007, −0.003
Group (Conventional Oxygenator: Ref. category)			
HLM Membrane Oxygenator	0.047	0.026	0.006, 0.088
ECLA Oxygenator	0.036	0.086	−0.005, 0.077
No treatment, No Ischemia	0.111	<0.001	0.07, 0.152

**Table 3:** Longitudinal analysis for all measurements for ECLA Oxygenator versus HLM Membrane Oxygenator.

LVSP	b	*p*	95% CI
Time	1.59	<0.001	1.045, 2.135
Group (HLM Membrane Oxygenator: Ref. category)			
ECLA Oxygenator	−0.028	0.988	−3.791, 3.735
LVEDP			
Time	0.096	0.516	−0.194, 0.387
Group (HLM Membrane Oxygenator: Ref. category)			
ECLA Oxygenator	0.255	0.803	−1.749, 2.259
LVDP			
Time	1.494	<0.001	0.776, 2.211
Group (HLM Membrane Oxygenator: Ref. category)			
ECLA Oxygenator	−0.283	0.911	−5.237, 4.671
HR			
Time	4.518	<0.001	2.556, 6.481
Group (HLM Membrane Oxygenator: Ref. category)			
ECLA Oxygenator	1.625	0.815	−11.978, 15.228
RPR			
Time	0.784	<0.001	0.418, 1.151
Group (HLM Membrane Oxygenator: Ref. category)			
ECLA Oxygenator	0.121	0.925	−2.408, 2.649
dPdT			
Time	23.028	0.063	−1.264, 47.321
Group (HLM Membrane Oxygenator: Ref. category)			
ECLA Oxygenator	−15.937	0.893	−247.514, 215.639
MdPdT			
Time	22.514	0.001	8.644, 36.384
Group (HLM Membrane Oxygenator: Ref. category)			
ECLA Oxygenator	4.281	0.931	−93.067, 101.63
CF			
Time	0.291	<0.001	0.222, 0.36
Group (HLM Membrane Oxygenator: Ref. category)			
ECLA Oxygenator	0.177	0.811	−1.274, 1.628
pH			
Time	−0.001	0.273	−0.003, 0.001
Group (HLM Membrane Oxygenator: Ref. category)			
ECLA Oxygenator	−0.011	0.612	−0.052, 0.031

**Table 4:** Longitudinal analysis for all measurements for KHB + Protein Solution versus KHB Solution.

LVSP	b	*p*	95% CI
Time	1.633	<0.001	1.101, 2.166
Group (KHB Solution: Ref. category)			
KHB + Protein Solution	4.617	0.014	0.94, 8.294
LVEDP			
Time	−0.016	0.913	−0.31, 0.277
Group (KHB Solution: Ref. category)			
KHB + Protein Solution	−2.294	0.027	−4.321, −0.266
LVDP			
Time	1.65	<0.001	0.951, 2.348
Group (KHB Solution: Ref. category)			
KHB + Protein Solution	6.911	0.005	2.091, 11.73
HR			
Time	4.793	<0.001	2.877, 6.709
Group (KHB Solution: Ref. category)			
KHB + Protein Solution	19.469	0.004	6.238, 32.7
RPR			
Time	0.874	<0.001	0.516, 1.233
Group (KHB Solution: Ref. category)			
KHB + Protein Solution	4.14	0.001	1.662, 6.618
dPdT			
Time	16.359	0.157	−6.289, 39.007
Group (KHB Solution: Ref. category)			
KHB + Protein Solution	337.583	0.002	120.471, 554.695
MdPdT			
Time	13.938	0.05	−0.002, 27.877
Group (KHB Solution: Ref. category)			
KHB + Protein Solution	315.312	<0.001	211.041, 419.584
CF			
Time	0.009	0.877	−0.105, 0.123
Group (KHB Solution: Ref. category)			
KHB + Protein Solution	5.573	<0.001	4.306, 6.84
pH			
Time	0.001	0.173	−0.001, 0.003
Group (KHB Solution: Ref. category)			
KHB + Protein Solution	0.047	0.027	0.005, 0.088

**Table 5:** Carbozen consumption of the evaluated oxygenation systems.

Oxygenator	Gas Flow (L/min)	Carbozen Consumption (3 h)	Nr of Experiments per Cylinder
Conventional Bubble	0.6	108 L	18
CAPIOX FX05	0.4	72 L	27
Novalung iLA	0.4	72 L	27

## Data Availability

The research data presented in this study are available on request from the corresponding authors.
